# Supplementary material for: Structure of AcMNPV nucleocapsid reveals DNA portal organization and packaging apparatus of circular dsDNA baculovirus
Source: Nat Commun. 2025 May 24;16:4844. doi: 10.1038/s41467-025-60152-2 (PMC12103608; doi:10.1038/s41467-025-60152-2)
Supplement: Supplementary file 1 — Supplementary Information [file 41467_2025_60152_MOESM1_ESM.pdf]

Structure of AcMNPV nucleocapsid reveals DNA portal organization and packaging apparatus of circular dsDNA baculovirus.

Gregory Effantin[1,*], Eaazhisai Kandiah[2,*] and Martin Pelosse[3,*]

Supplementary information

Supplementary Figure 1

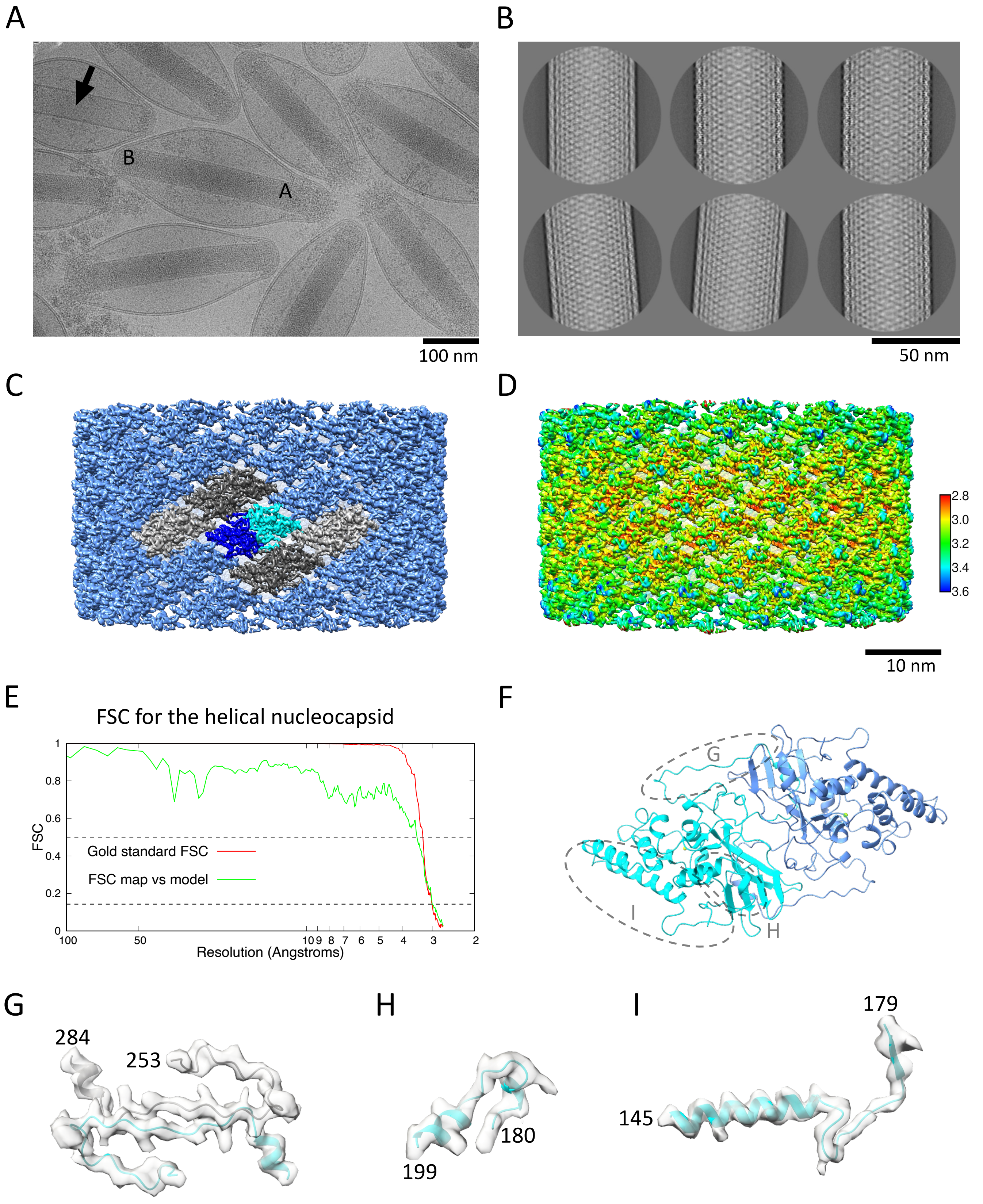

Supplementary Figure 1: 3D structure of the helical capsid of AcMNPV.

A- Electron micrograph of AcMNPV purified BV particles. Empty nucleocapsid, apical cap and basal structure are labelled by black arrow, A and B respectively. B- 2D classes of the helical capsid formed by VP39. C- Isosurface representation of the 3D reconstruction of the AcMNPV helical capsid. One dimer of VP39 is highlighted in cyan/dark blue. The assembly formed by VP39 follows a C14 symmetry combined with a helical symmetry. Two symmetric dimers flanking the highlighted dimer are colored in light or dark gray for C14 or helical symmetry, respectively. D- Isosurface representation of the 3D reconstruction of the AcMNPV helical capsid colored by local resolution. E- Fourier Shell Correlation (FSC) curves for the helical capsid of AcMNPV. The gold standard FSC between two independent 3D reconstructions is shown in red while the FSC curve between the cryo-EM coulomb potential map and the corresponding refined atomic model is in green. The dotted horizontal lines represent FSC=0.143 and 0.5 which are used as cutoffs to determine the resolutions for the "Gold standard FSC" and the "FSC map vs model" respectively. F- Ribbon representation of a VP39 dimer (color code same as C). The Zn ion in each monomer is also represented. G to I - Illustrations of the 3D reconstruction and atomic model quality of AcMNPV helical capsid. The coulomb potential map from cryo-EM is in transparent grey. G- Zoomed view on the region highlighted in panel (F). Two helically related dimers interact laterally through loops formed by residues 253 to 284. H- Zoomed view on the region highlighted in panel (F). Residues 180 to 199 of VP39 are shown, they are located at the surface of the VP39 dimer. The same region is implicated with Ac104 in the apical and basal caps. I- Zoomed view on the region highlighted in panel (F). Residues 145 to 179 of VP39 are shown in their conformation in the helical capsid. The same region adopts a different conformation in the VP39 subunit which interacts with Ac109 in the apical cap and the basal structure.

Supplementary Figure 2

Flowchart for the preprocessing and image analysis of the helical capsid

41 386 movies → 37 453 micrographs

- Motion correction
- CTF estimation
- Select CTF max res < 6Å & Defocus > 4000 Å

37 453 micrographs →

- Select 1064 micrographs
- Manual helical picking
- Class 2D
- Get 1st templates for capsid and basal/apical ends

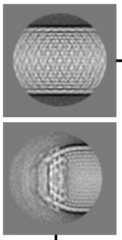

To Supplementary Fig. 3

Structure of the helical capsid

- Expand to 7593 micrographs
- Autopick with template
- Class2D 3x

48 565 particles

- 3D refinement with a cylinder as template and C14 symmetry
- Fit of alphafold VP39 dimers
- Initial helical parameters from fitted VP39s (z=42.6Å, Φ=-7°)
- 3D refinement

4.1 Å resolution (Nyquist)

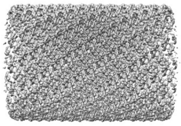

- Expand to 15 751 micrographs<br>- Template autopicking	- Add another 19 459 micrographs<br>- Template autopicking
612 435 particles	598 364 particles
- Class2D	- Class2D x2
545 747 particles	419 919 particles
- 3D refinement<br>- 3D classification, 5 classes	- 3D refinement<br>- 3D classification, 5 classes
182 474 particles	144 622 particles
- Extract bin 1<br>- 3D refinement<br>- 3D classification, 3 classes	- Extract bin 1<br>- 3D refinement<br>- 3D classification, 3 classes
84 446 particles	138 856 particles
x2 [- 3D refinement<br>- CTF refinement<br>- 3D refinement]<br>- Bayesian polishing<br>- 3D refinement	x2 [- 3D refinement<br>- CTF refinement<br>- 3D refinement]<br>- Bayesian polishing<br>- 3D refinement

223 302 particles

- 3D refinement
- 3D classification, 5 classes

138 225 particles

- 3D refinement

3.0 Å resolution

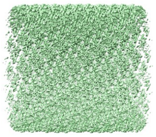

Supplementary Fig. 1

Supplementary Figure 2: SPA image processing workflow for the helical capsid sheath.

Flowchart outlining the single particle image analysis pipeline for the helical capsid sheath, including motion correction, CTF estimation, particle picking, 2D and 3D classification and refinement performed using RELION. Helical parameters are indicated within the flowchart. Downstream processing steps shown in other figures are marked with green text boxes. Final 3D reconstructions obtained in this flowchart are highlighted by red rectangles, along with their corresponding figure references.

Supplementary Figure 3

Flowchart for the image analysis of the basal structure

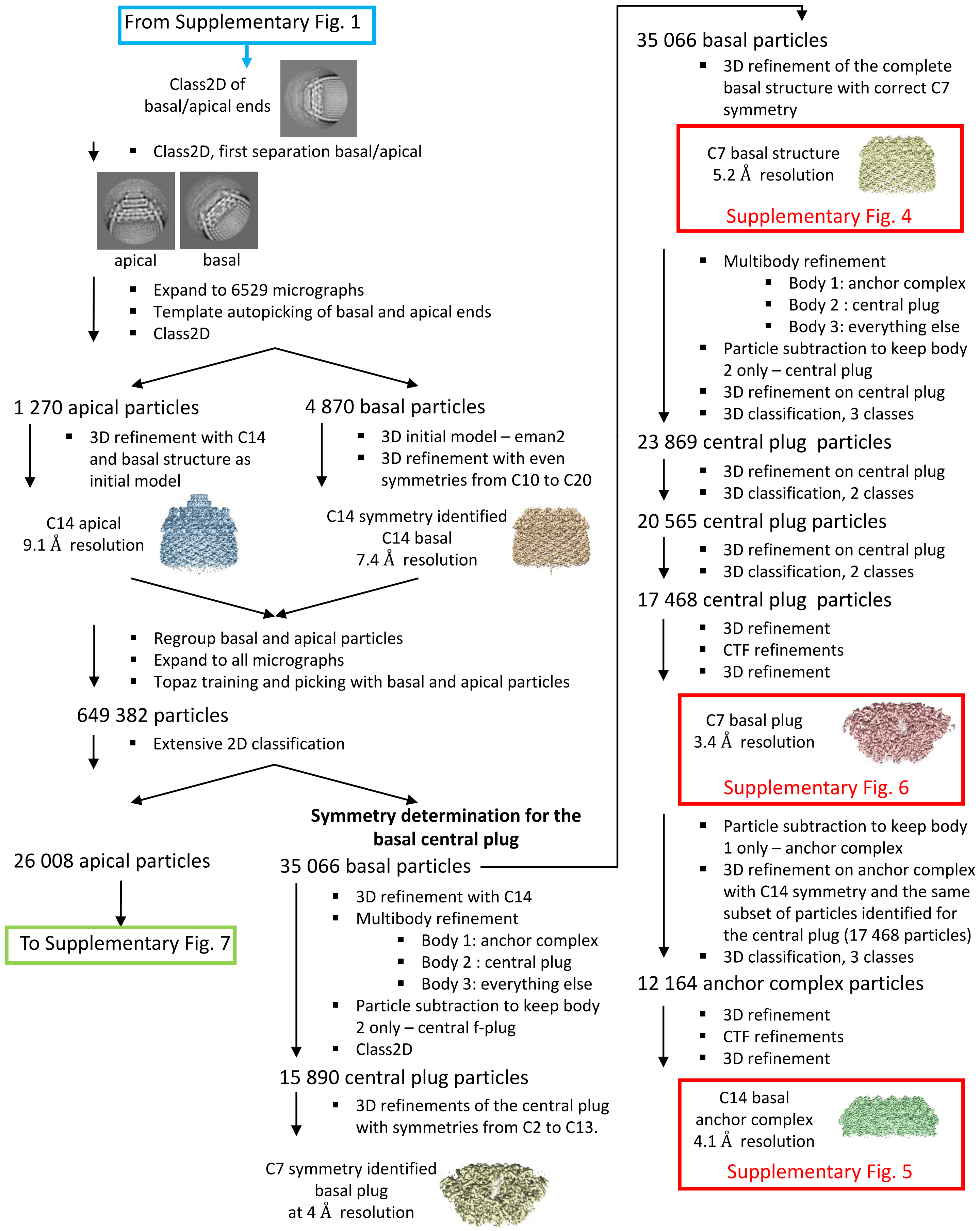

Supplementary Figure 3: Cryo-EM image processing workflow for the basal structure.

Flowchart outlining the image analysis pipeline followed primarily for the basal structure. Note that both apical and basal ends were treated together for 2D classification starting from the pre-processed micrographs explained in Supplementary Figure 2. A blue text box denotes continuation from the previous flowchart figure. Downstream processing steps shown in other figures are marked with green text boxes. Final 3D reconstructions obtained in this flowchart are highlighted by red rectangles, along with their corresponding figure references.

Supplementary Figure 4

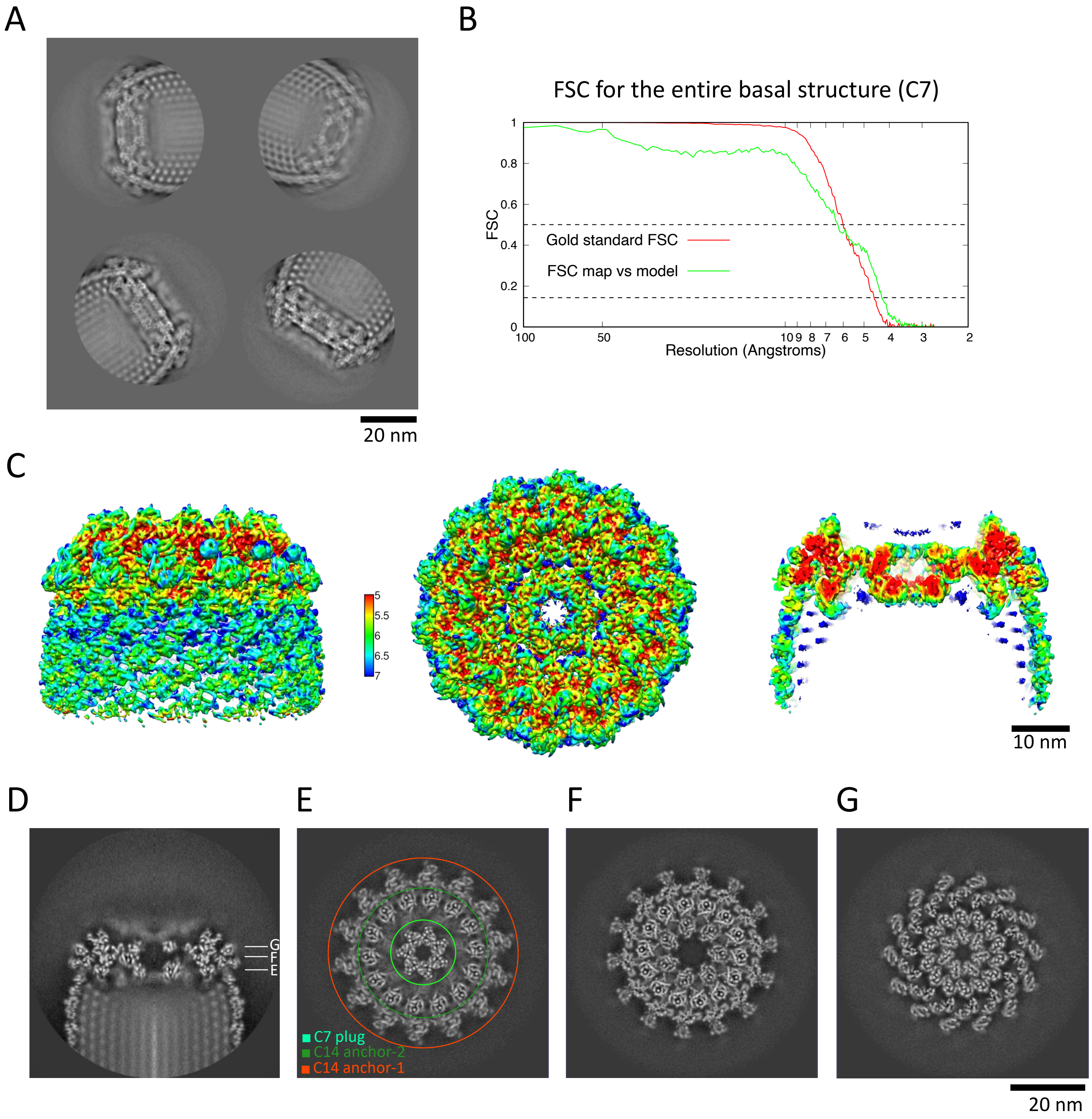

Supplementary Figure 4: 3D structure of the entire basal structure of AcMNPV.

A- 2D classes of the basal structure. B- Fourier Shell Correlation (FSC) curves for the entire basal structure. The gold standard FSC between two independent 3D reconstructions is shown in red while the FSC curve between the cryo-EM coulomb potential map and the corresponding refined atomic model is in green. The two dotted horizontal lines represent FSC=0.143 and 0.5 which are used as cutoffs to determine the resolutions for the “Gold standard FSC” and the “FSC map vs model” respectively. C- Isosurface representation of the 3D reconstruction obtained for the AcMNPV entire basal structure colored by local resolution (side view (left), top view (middle), cut-away side view (right). D- Central section through the 3D reconstruction of the basal structure (xz plane). The z heights of the perpendicular sections (xy plane) shown in E to G are indicated. E to G- Sections (in xy plane) through the basal structure 3D map highlighting the relative arrangement of the C7 plug into the C14 anchor complex.

Supplementary Figure 5

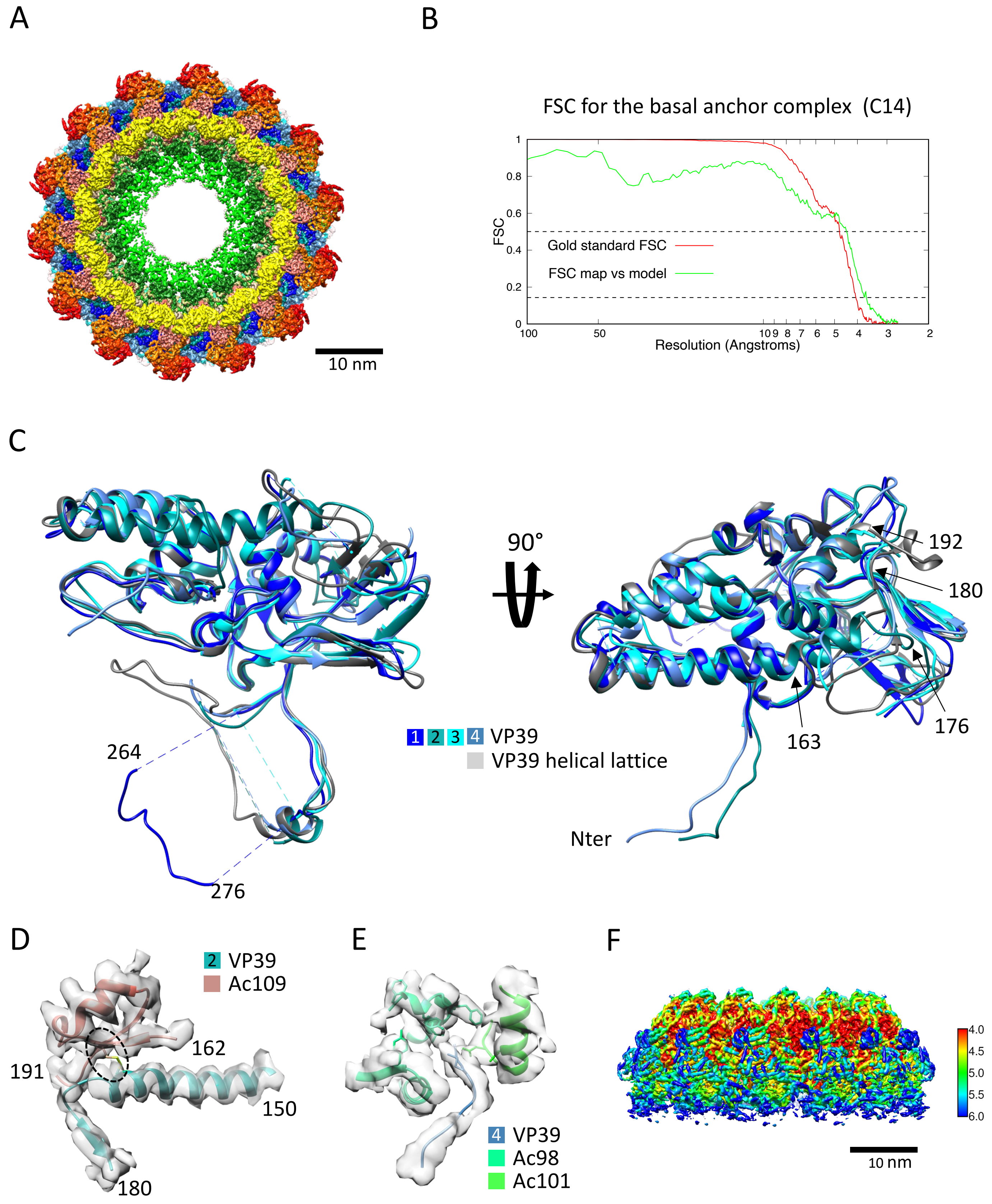

Supplementary Figure 5: 3D structure of the anchor complex of the basal structure of AcMNPV.

A- Isosurface representation of the 3D reconstruction (top view) of the C14 anchor-1/anchor-2 complex colored by its protein composition according to the color code of Fig. 2. B- Fourier Shell Correlation (FSC) curves for the AcMNPV basal anchor complex. The gold standard FSC between two independent 3D reconstructions is shown in red while the FSC curve between the cryo-EM coulomb potential map and the corresponding refined atomic model is in green. The dotted horizontal lines represent FSC=0.143 and 0.5 which are used as cutoffs to determine the resolutions for the "Gold standard FSC" and the "FSC map vs model" respectively. C- Superposition of VP39 monomers from the basal structure (VP39_1 to _4) and the helical capsid (VP39_helical lattice). Two views highlight the important conformational differences: residues 264 to 276 (VP39_1 vs VP39 helical lattice), N terminal region of both VP39_2 and VP39_4 have an extended conformation versus the others, residues 163 to 176 where VP39_2 has an extended α-helix compared to the others because of its binding to Ac109 and residues 180 to 192 where VP39_1 to _4 have a different conformation compared to VP39 in the helical lattice because of the binding of Ac104 in the anchor-1 complex of both the basal structure and apical cap. D, E - Illustrations of the 3D reconstruction and atomic model quality of the basal anchor complex. The coulomb potential map is in transparent grey. D- Zoomed view of Ac109binding on top of the VP39 lattice in both basal and apical caps inducing a conformational change in residues 163 to 176 of VP39_2 which then allows the formation of a disulfide bond between VP39_2 C169 and Ac109 C187 (dotted lines). E- Region represented in Fig. 2F where the VP39_4 N terminus from anchor-1 of au_i interacts with Ac98 from anchor-2 of au_i and the lower Ac144/Ac101 module of anchor-2 of au_{i-1}. Side chains are shown for residuespredicted to form hydrogen bonds (M8 for VP39_4, L32 and Y40 for Ac98 and, D273 and N279 for Ac102). F- Isosurface representation of the 3D reconstruction obtained for the basal C14 anchor complex colored by local resolution.

Supplementary Figure 6

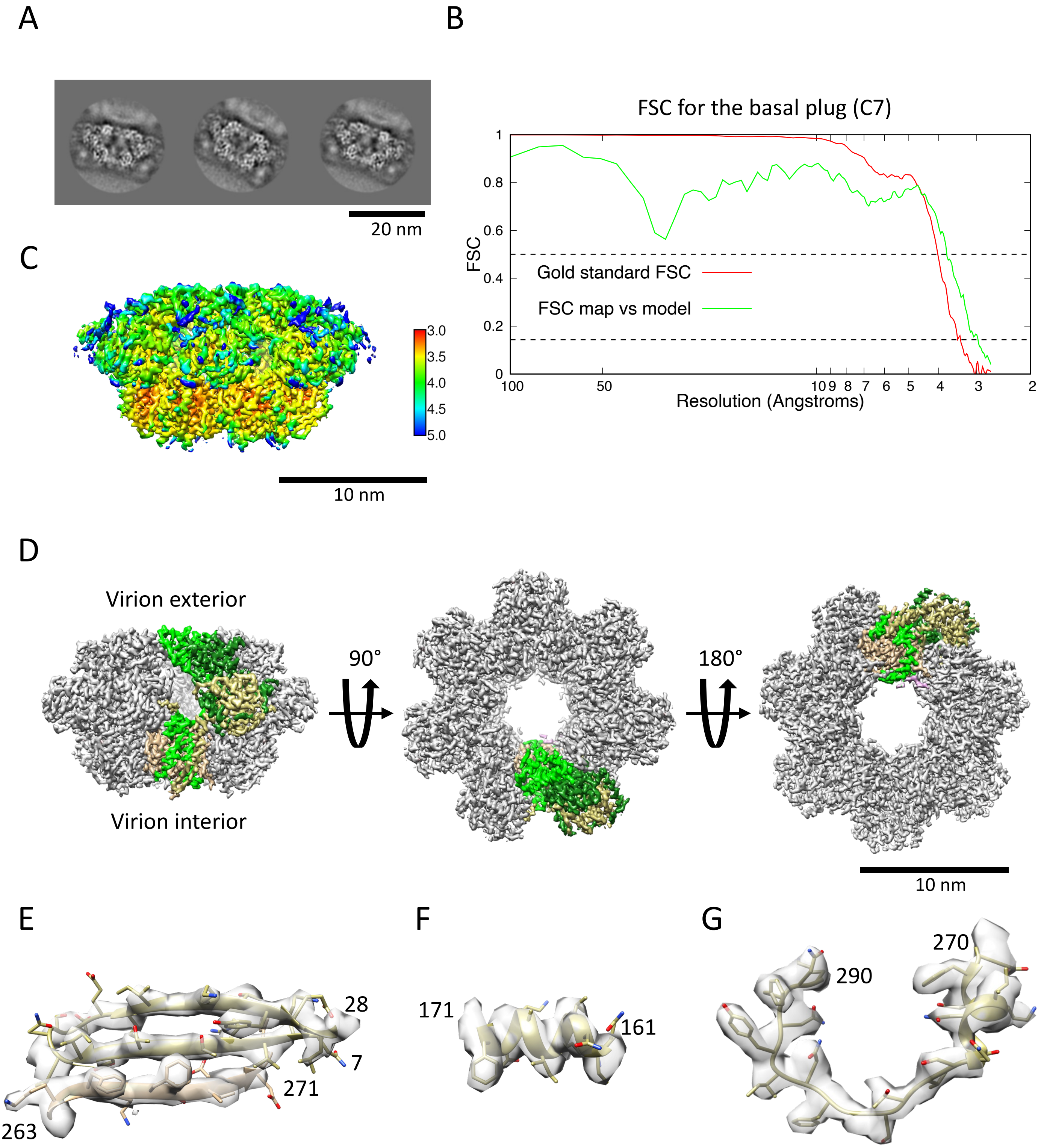

Supplementary Figure 6: 3D structure of the basal C7 plug of AcMNPV.

A- 2D classes of the basal C7 plug after density subtraction. B- Fourier Shell Correlation (FSC) curves for the basal C7 plug. The gold standard FSC between two independent 3D reconstructions is shown in red while the FSC curve between the cryo-EM coulomb potential map and the corresponding refined atomic model is in green. The two dotted horizontal lines represent FSC=0.143 and 0.5 which are used as cutoffs to determine the resolutions for the "Gold standard FSC" and the "FSC map vs model" respectively. C- Isosurface representation of the 3D reconstruction (side view) of the basal C7 plug colored by local resolution. D - Isosurface representation of the cryo-EM 3D reconstruction of the C7 plug viewed from 3 different orientation (side view (left), top view from the outside (middle) and the inside (right)). In each panel, the 4 proteins (2 copies each of Ac101 and Ac144) composing an asymmetric unit are colored differently (using the same color code as in Figure 3). E to G- Illustrations of the quality of the 3D reconstruction and atomic model fit for the basal C7 plug. The coulomb potential map from cryo-EM is in transparent grey. E- Relates to Figure 3H: The N-terminus of the upper Ac144_1 (residues 7 to 28) interacts with the C-terminal (residues 263 to 271) of the lower Ac144_2 by β-sheet augmentation. F- Relates to Figure 3G: residues 161 to 171 from Ac144_1 are shown with side chains. G- Relates to Figure 3I: the C-terminus of Ac144_1 of the upper module (residues 270 to 290) is shown with side chains depicted.

Supplementary Figure 7

Flowchart for the image analysis of the apical cap – part 1

From Supplementary Fig. 3

26 008 apical particles

- 3D refinement with C14
- Multibody refinement
    - Body 1: anchor complex
    - Body 2 : central plug
    - Body 3: crown-like ring

Symmetry determination and 3D structure or the crown

- Particle subtraction to keep body 3 only, the crown-like ring
- Class2D

21 190 crown-like particles

- 3D refinements of the crown-like ring with symmetries from C2 to C30.

C21 symmetry identified
Crown at 4.2 Å resolution

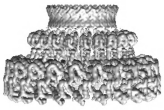

- 3D refinement on C21 crown
- 3D classification, 3 classes

6 390 crown-like particles

- 3D refinement
- CTF refinements
- 3D refinement

C21 crown
3.5 Å resolution

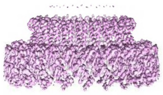

Supplementary Fig. 12

Symmetry determination for the central plug

- Particle subtraction to keep body 2 only, the central plug
- Class2D

12 981 central plug particles

- 3D classification, C1, 3 classes: 1 3D class has C2 symmetry

12 213 central plug particles

- 3D refinement, C2 symmetry

C2 symmetry identified
Central plug
at 8.3 Å resolution

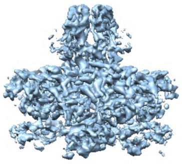

3D reconstruction of the C2 apical core (anchor complex + C2 plug)

26 008 apical particles

- Particle subtraction to keep C2plug + anchor-2
- 3D refinement with C2 symmetry
- Particle subtraction to keep C2plug + anchor-1 & -2
- 3D refinement with C2 symmetry

C2 apical core
6.0 Å resolution

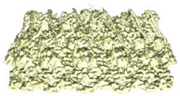

Supplementary Fig. 9

To Supplementary Fig. 8

Supplementary Figure 7: Cryo-EM image processing workflow for the apical cap-part 1.

Flowchart outlining the image analysis pipeline followed for the apical cap, including the steps followed for the apical C21 crown ring and the C2 central plug. A blue text box denotes continuation from the previous flowchart figure. Downstream processing steps shown in other figures are marked with green text boxes. Final 3D reconstructions obtained in this flow are highlighted by red rectangles, along with their corresponding figure references.

Supplementary Figure 8 **Flowchart for image analysis of the apical cap – part 2**

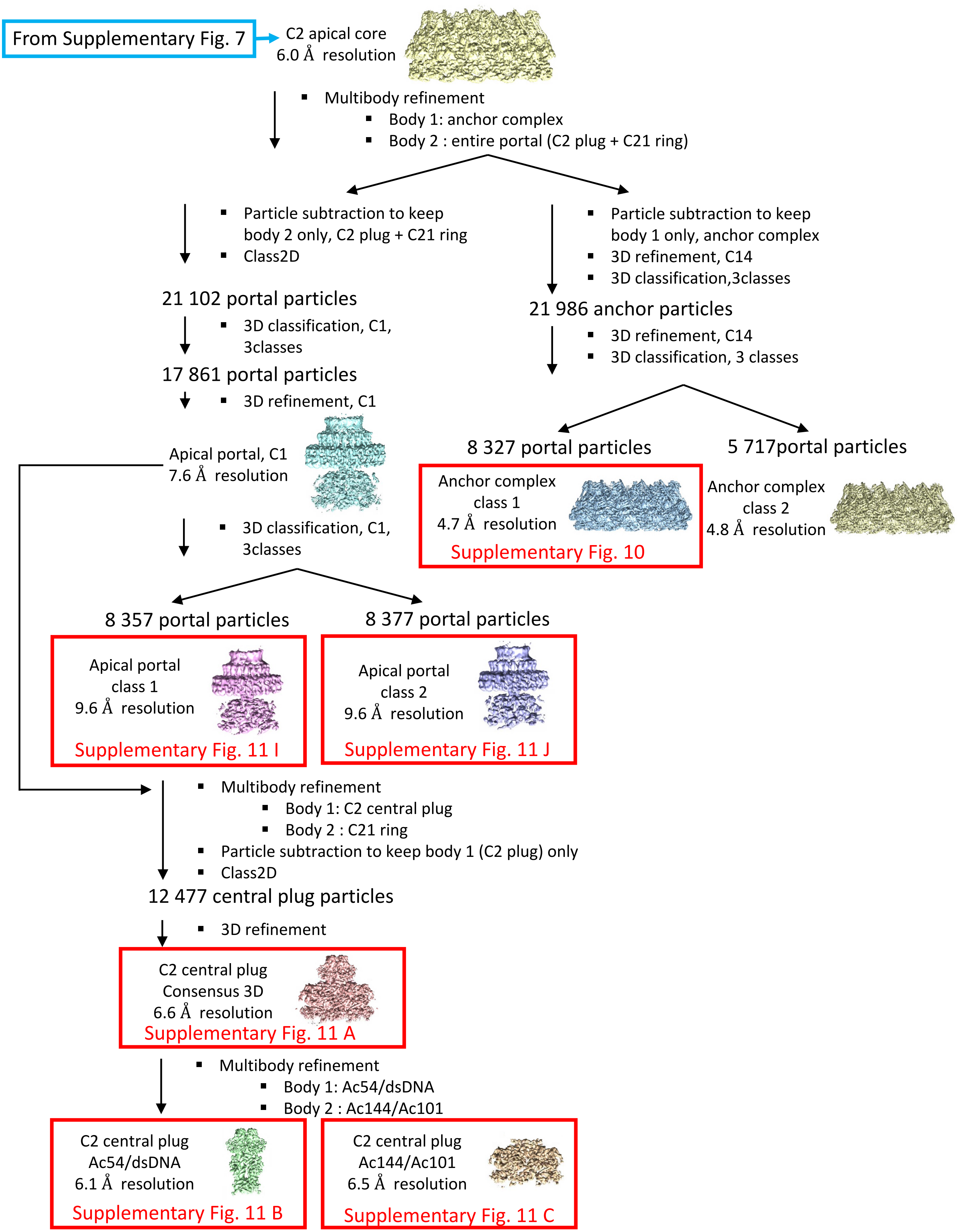

Supplementary Figure 8: Cryo-EM image processing workflow for the apical cap-part 2.

Flowchart outlining the image analysis pipeline followed for the apical cap, including the steps followed for the C2 plug, C2/C21 portal and apical C14 anchor complex structures. A blue text box denotes continuation from the previous flowchart figure. Final 3D reconstructions obtained in this flow are highlighted by red rectangles, along with their corresponding figure references.

Supplementary Figure 9

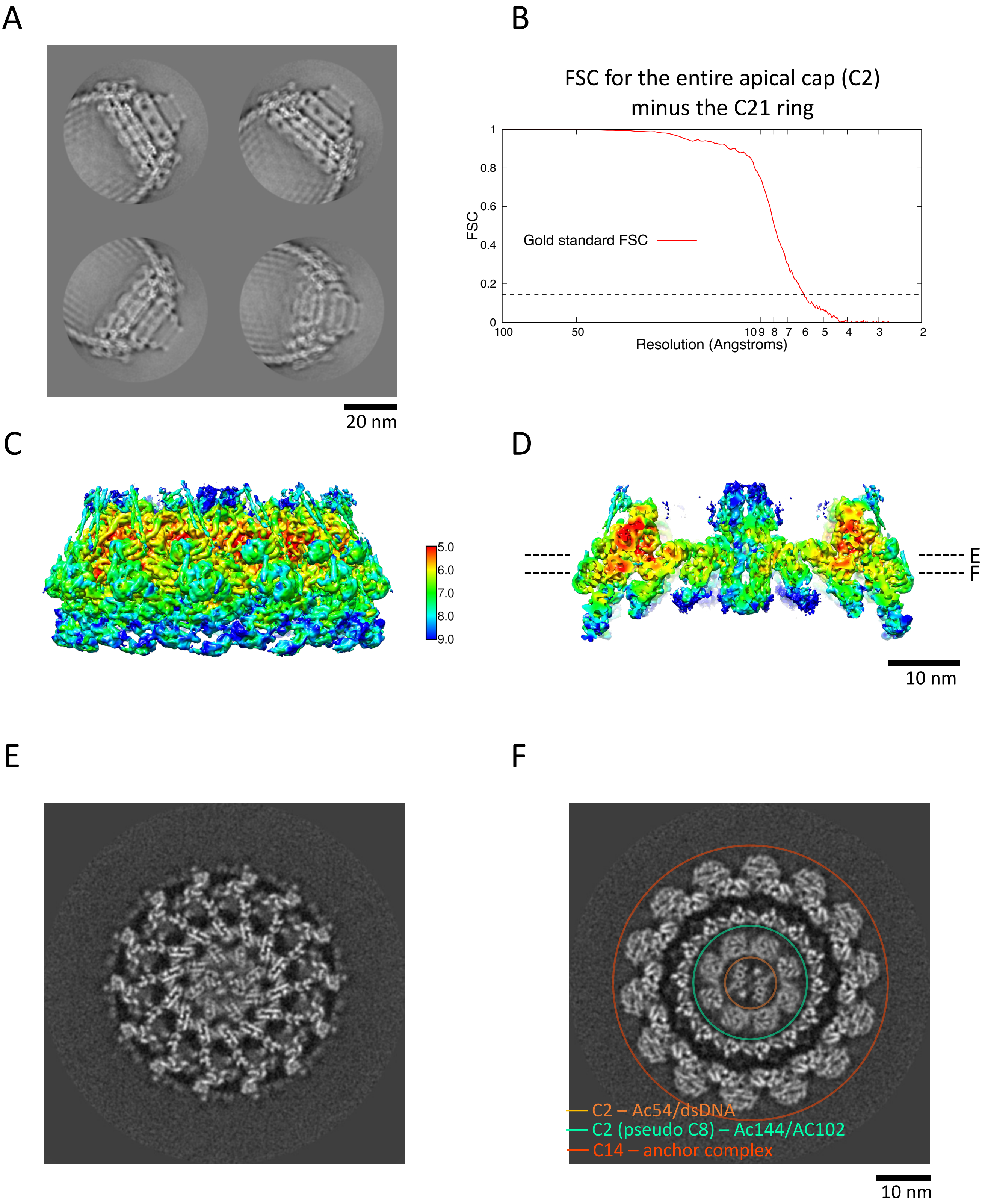

Supplementary Figure 9: 3D structure of the C2 part of the apical cap of AcMNPV.

A- 2D classes of the entire apical cap of. B- Fourier Shell Correlation (FSC) curve for the part of the apical cap which has C2 symmetry (excluding the C21 ring). The gold standard FSC between two independent 3D reconstructions is shown in red. The dotted horizontal line represents FSC=0.143 which is used as a cutoff to determine the resolution for the “Gold standard FSC”. C - Isosurface representation of the C2 apical cap showing only the assemblies exhibiting C2 symmetry colored by local resolution, (resolution scale in Å is indicated). D- same as (C) but shown as a sliced view to visualize the interior. E & F- Sections (in xy plane, their respective z height is indicated on panel (D)). These two panels highlight the interconnection between the C2 plug (comprising Ac54/dsDNA) and its surrounding shell of Ac144/Ac101, arranged as an octameric assembly of two Ac144/Ac101 modules. This shell is embedded within the C14 symmetric anchor-2 complex, which also consists of repeated Ac144/Ac101 module pairs). The boundary between these different assemblies is delineated in panel F.

Supplementary Figure 10

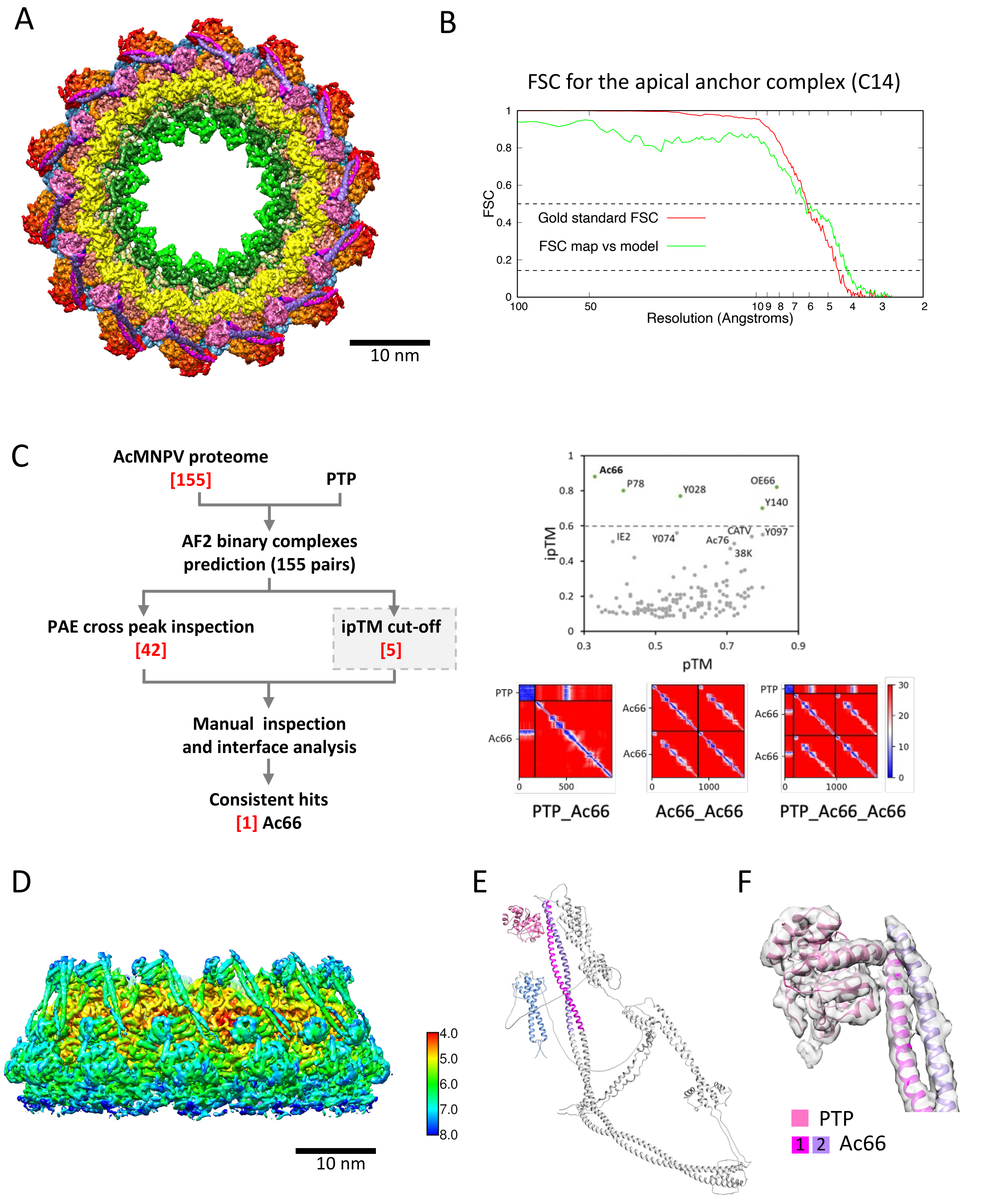

Supplementary Figure 10: 3D structure of the apical anchor complex of AcMNPV.

A- Isosurface representation of the 3D reconstruction (top view) of the apical C14 anchor-1/anchor-2 complex colored by its protein composition according to the color code of Figure 2. B- Fourier Shell Correlation (FSC) curves for the anchor complex of the apical cap. The gold standard FSC between two independent 3D reconstructions is shown in red while the FSC curve between the cryo-EM coulomb potential map and the corresponding refined atomic model is in green. The two dotted horizontal lines represent FSC=0.143 and 0.5 which are used as cutoffs to determine the resolutions for the "Gold standard FSC" and the "FSC map vs model" respectively. C- Identification of Ac66 as the PTP interacting partner. Left: Workflow of the *in-silico* protein-protein interaction screen used in this study. Upper right: Predicted template modeling score (pTM) and interface predicted template modeling score (ipTM) for PTP-baculoviral proteins pairs analyzed with AlphaFold multimer v2.3. Top hits, colored in green, were manually inspected for interface analysis. Lower right: PAE (Predicted Alignment Error) plot of the AlphaFold multimer prediction of the binary interaction between PTP and Ac66, Ac66 dimer and PTP and Ac66 dimer. D- Isosurface representation of (A) but shown as a side view and colored by local resolution (resolution scale in Å is indicated). E- Alphafold prediction of PTP in complex with Ac66 dimer. PTP is colored in pink. Ac66 dimer is colored in gray except the regions which have been identified in anchor-1 (residues 310 to 386 – colored in shades of violet and in the C21 ring (residues 1 to 75- colored in blue). F- The fit of PTP/Ac66 dimer into the EM coulomb potential 3D map (in transparent gray) is shown to highlight the confidence in the identification of PTP and Ac66 dimer in that region of the apical cap.

Supplementary Figure 11

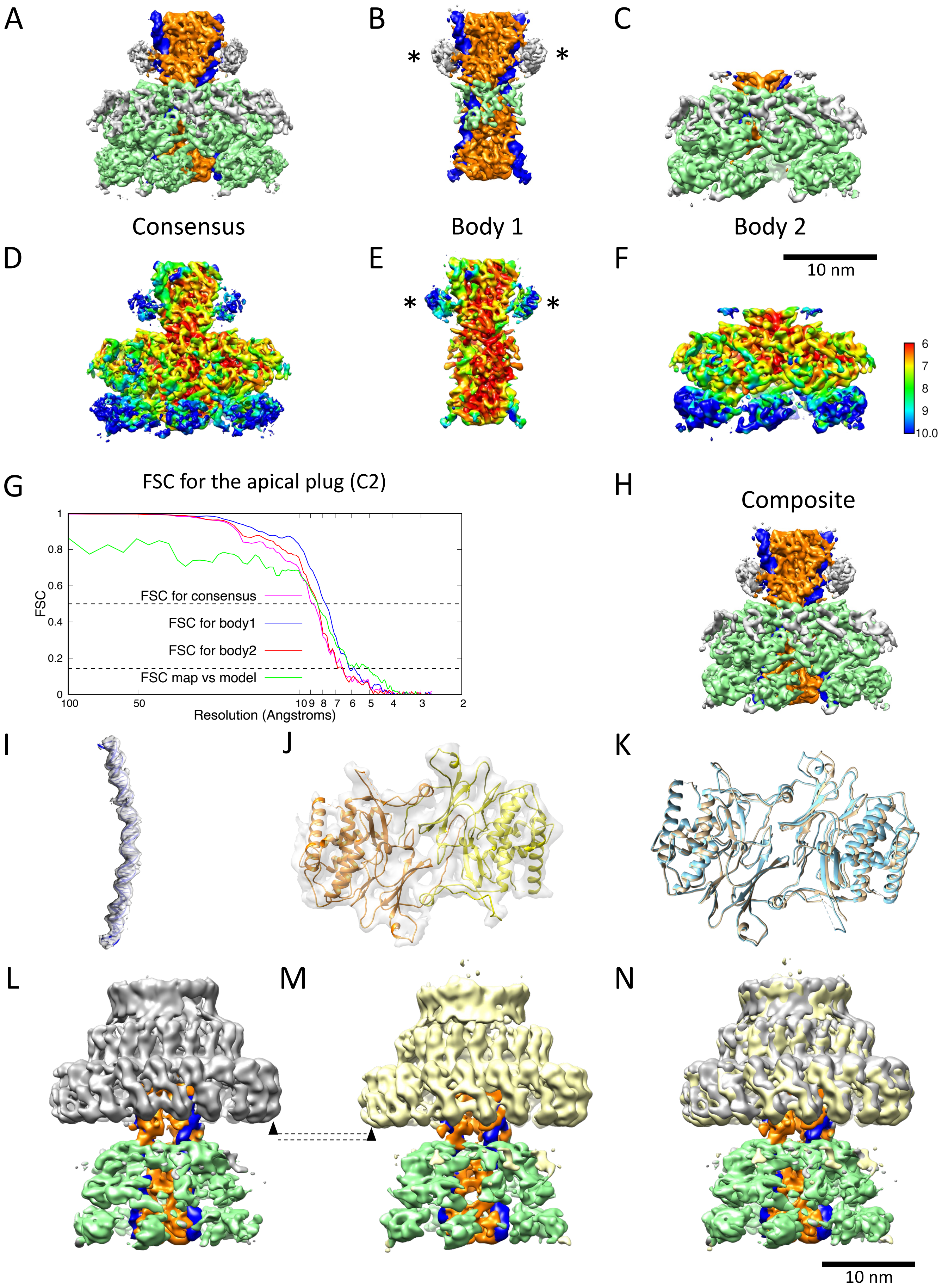

Supplementary Figure 11: 3D structure of the apical central plug of AcMNPV.

A- Isosurface representation (side view) of the 3D consensus map of the apical C2 plug showing protein/DNA composition (same color code as in Fig. 1). B, C- Isosurface representation (side view) of the two focused 3D maps (body 1 (B) and body 2 (C)) that together form the composite map shown in (H). The (*) in (B) indicate un-assigned densities near the dsDNA and Ac54-1 dimer. D-F- Same as A-C but colored by local resolution (resolution scale in Å is indicated). G- Fourier Shell Correlation (FSC) curves for the C2 plug of the apical cap. The gold standard FSC between two independent 3D reconstructions for the consensus (A), body 1 (B) and body 2 (C) are shown in violet, blue and red respectively while the FSC curve between the composite map (H) and the corresponding refined atomic model is in green. The dotted horizontal lines represent FSC=0.143 and 0.5 which are used as cutoffs to determine the resolutions for the "Gold standard FSC" and the "FSC map vs model" respectively. H- Isosurface representation of the 3D composite map (side view) of the apical C2 plug colored according to its protein composition using the color code of Fig. 1. I- View showing the fitted model of the dsDNA into the cryo-EM map, shown as a transparent grey isosurface. J- View of an Ac54 dimer (monomers colored orange and yellow respectively) fitted into the cryo-EM map. K- Superposition of the two Ac54 dimers that compose the asymmetric unit of the apical C2 plug. The best superposition with lowest RMSD is achieved with protomer 4 superposed on protomer 1. L and M- Asymmetric (C1) 3D reconstructions of two apical DNA portal classes, each containing a C2 plug (Ac54/dsDNA/Ac101/Ac144, colored as in A) and a C21 ring (grey in L and yellow in M). The maps are aligned through the C2 plug to highlight differences in C21 ring position. The two arrowheads points to the difference in height between the 2 classes. N- Superimposition of the two 3D reconstructions shown in panels L and M.

Supplementary Figure 12

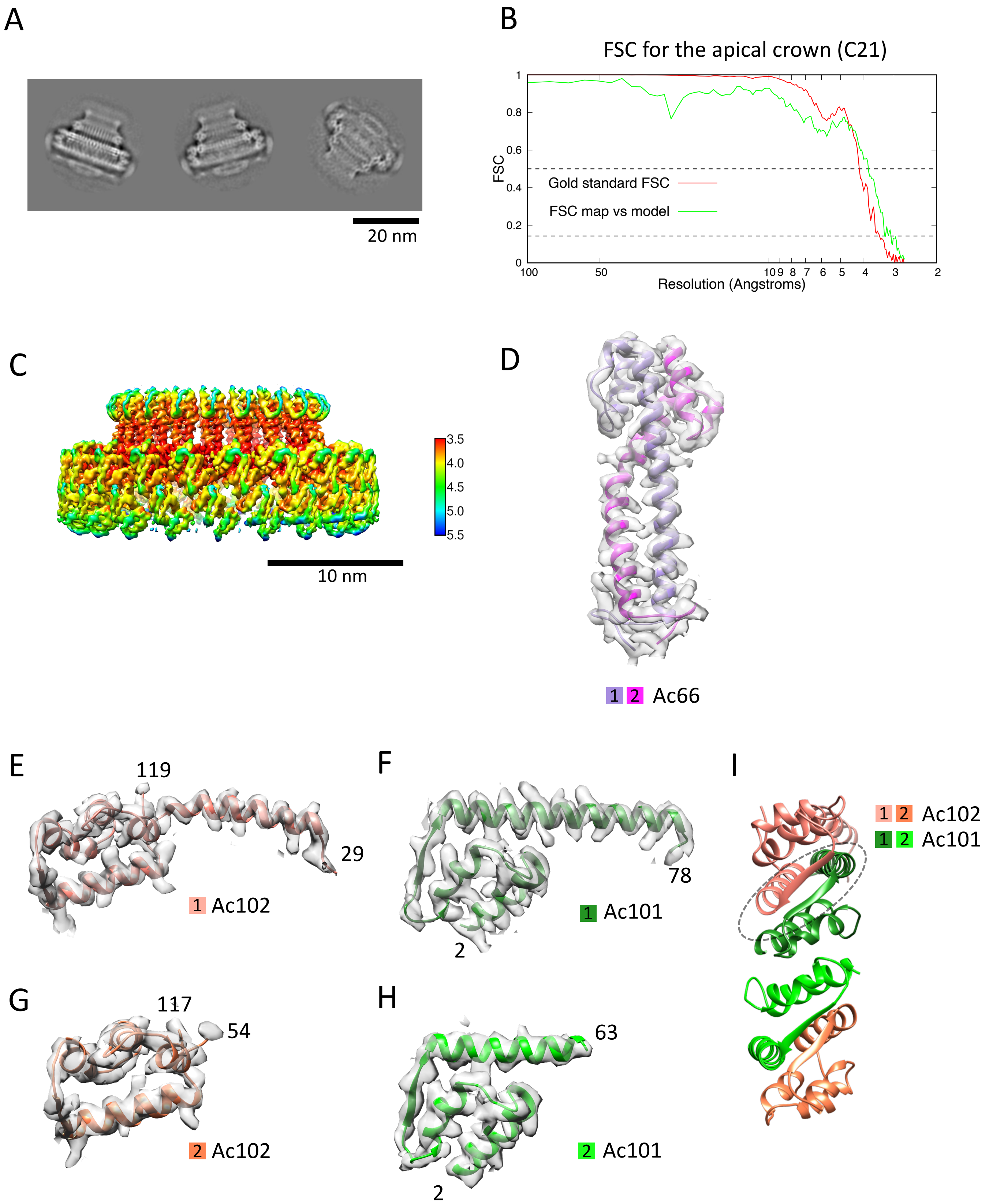

Supplementary Figure 12: 3D structure of the apical C21 ring of AcMNPV.

A- 2D classes of the C21 ring of the apical cap after density subtraction. B- Fourier Shell Correlation (FSC) curves for the C21 ring of the apical cap. The gold standard FSC between two independent 3D reconstructions is shown in red while the FSC curve between the cryo-EM coulomb potential map and the corresponding refined atomic model is in green. The two dotted horizontal lines represent FSC=0.143 and 0.5 which are used as cutoffs to determine the resolutions for the "Gold standard FSC" and the "FSC map vs model" respectively. C- Isosurface representation of the 3D reconstruction (side view) of the apical C21ring colored by local resolution (resolution scale in Å is indicated). D to H- Atomic models of the proteins composing the asymmetric units of the C21 ring fitted in their corresponding cryo-EM density map. Color code is the same as in Figure 5. I- View of the hetero-dimer formed by the two sets of Ac101 Nter/Ac102 composing the C21 ring. The intermolecular β-sheet between a Ac101/1c102 pair is highlighted with dotted lines.

Supplementary Figure 13

A

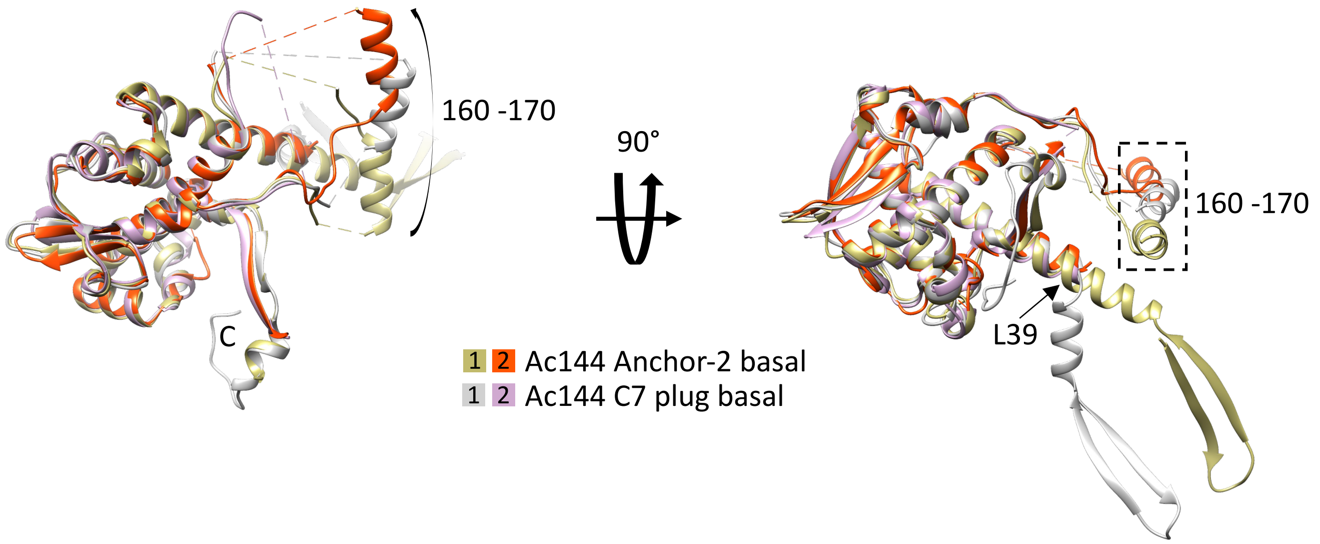

B

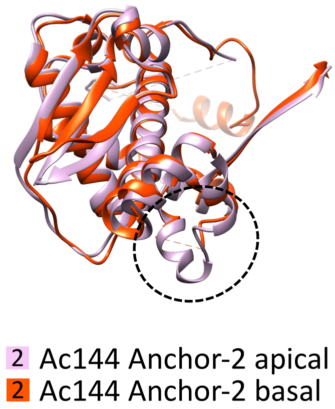

Supplementary Figure 13 Structural superposition of Ac144 from different anchor complexes.

A - Two different views of the superposition of Ac144_1 and Ac144_2 from the basal anchor-2 and C7 plug. The core of all Ac144s is nearly identical. The α-helix (residues 160 to 170) of all Ac144s (except Ac144_2 of the C7 plug) is involved in inter subunit interaction and has variable positions. Ac144_1 of the C7 plug also has a longer C-terminus (labelled C in the left panel) than the other subunits. The kink in the long α-helix of Ac144_1 of the C7 plug at L39 residue is indicated in the right panel. B- Superposition of Ac144_2 from the anchor-2 complex of either the basal structure or the apical cap. The dotted line highlights the region where Ac98 binds to Ac144_2 in the basal cap. In the apical cap, Ac144_2 has an extra small α-helix (residues 249 to 259) in that region.

Supplementary Figure 14

A

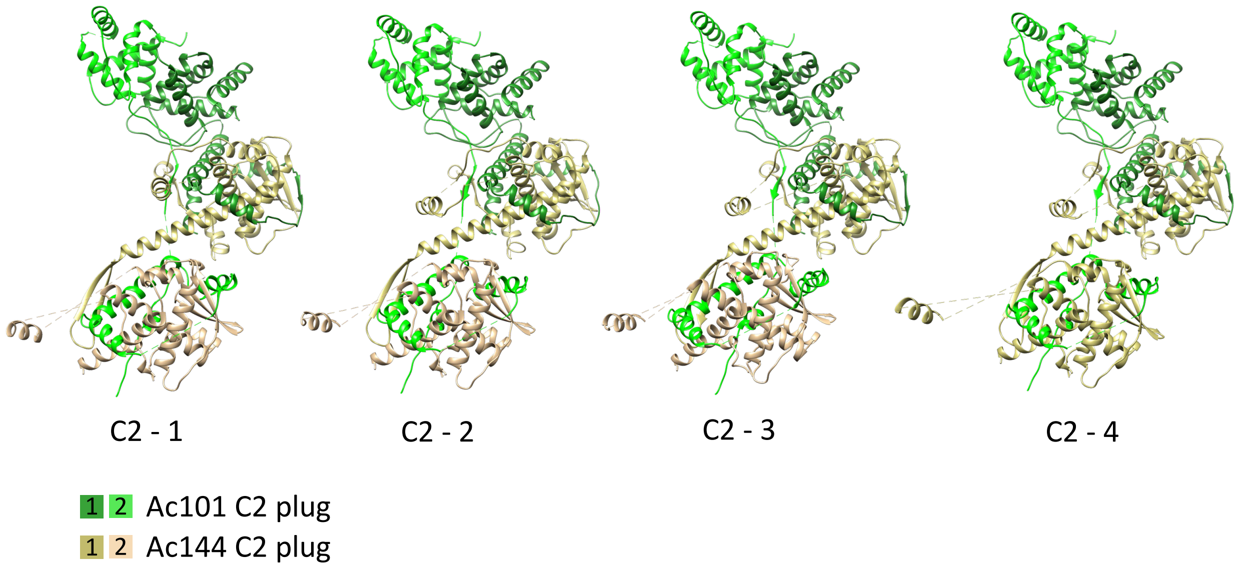

B

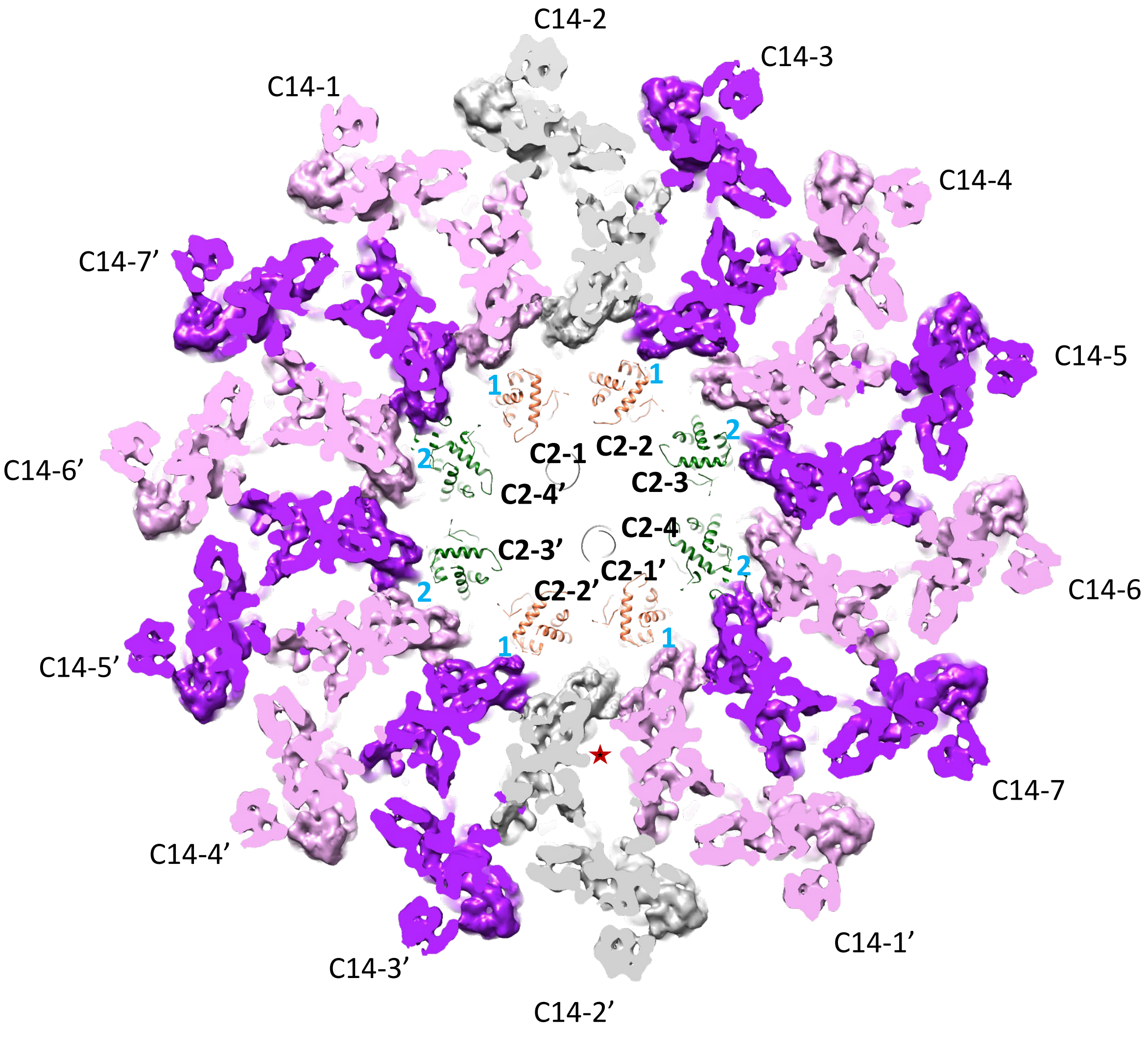

Supplementary Figure 14: Symmetry mismatch between C14 anchor ring to C2 plug of the apical cap.

A- View of the 4 equivalent (but not identical) assemblies (C2-1 to -4) that form the asymmetric unit of the C2 plug at the center of the apical cap. Each assembly is composed by two hetero dimers of Ac101/Ac144. B- Shown here is the C14 to C2 (pseudo-C8) symmetry mismatch in the apical cap between the anchor complex and the C2 plug. For representation purposes, electron density for C14 ring and atomic model for C2 ring are used. Protomers of Ac101/Ac144 proteins (C14-1 to C14-7) of the C14 ring and their C2-counterparts (C14-1' to C14-7') are marked. Contacts between C14 and C2 subunits are calculated using Chimera. Protomers that make extensive contacts (more than 50 contacts) are colored in dark purple and those that make fewer contacts are colored in light pink. Protomer C14-2 and C14-2' which does not make any contact with C2 Ac101/Ac144 protomers are in grey. Protomers of Ac101/Ac144 proteins (C2-1 to C2-4) of the C2 ring and their C2-counter parts (C2-1' to C2-4') are marked. Protomers C2-1 and C2-2 that are in contact with only one C14 protomer each are colored in coral while protomers C2-3 and C2-4 that are in contact with two C14 protomers each are colored in green. The number of C14 protomers that each C2 protomer is in contact with are also noted (blue numbers).

Supplementary Figure 15

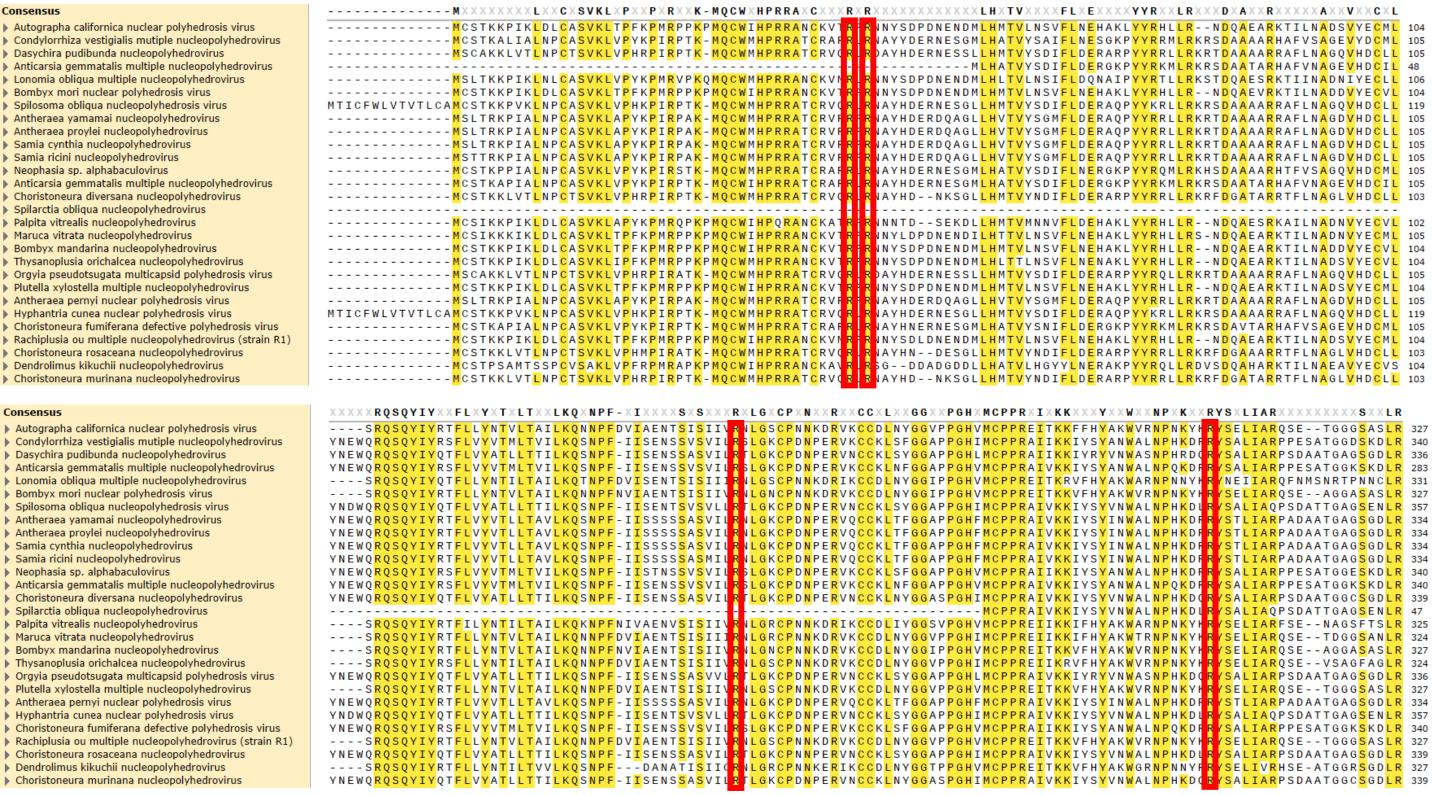

Supplementary Figure 15: Multiple sequence alignment (MSA) of Ac54 homologs from Alphabaculoviruses.

Alphabaculovirus homologs of Ac54 from *Autographa californica* nuclear polyhedrosis virus (AcMNPV) were subjected to MUSCLE[83] for MSA. Arginine residues identified in this study as critical for DNA binding at the baculovirus portal are seen to be conserved (red boxes).

Supplementary Table 1: Cryo-EM data collection, refinement and validation statistics

	#1 Helical VP39 (EMDB-51771) (PDB 9H1S)	#2 Basal structure - complete (EMDB-51791) (PDB 9H2A)	#3 Basal structure – anchor complex (EMDB-51792) (PDB 9H2B)
**Data collection and processing**			
Magnification	64 000	64 000	64 000
Voltage (kV)	300kV	300kV	300kV
Electron exposure (e–/Å^2)	30	30	30
Defocus range (μm)	-1 to -2.2	-1 to -2.2	-1 to -2.2
Pixel size (Å)	1.35	1.35	1.35
Symmetry imposed	C14/helical	C7	C14
Final particle images (no.)	138 225	35 066	12 164
Map resolution (Å)	3.0	5.2	4.1
FSC threshold	0.143	0.143	0.143
**Refinement**			
Model resolution (Å)	3.5	6.3	4.4
FSC threshold	0.5	0.5	0.5
Model composition			
Non-hydrogen atoms	4996	66 809	29 809
Protein residues	616	8142	3633
Ligands	2	8	4
R.m.s. deviations			
Bond lengths (Å)	0.0139	0.0043	0.0041
Bond angles (°)	1.35	0.93	0.91
Validation			
MolProbity score	1.6	2.0	1.8
Clashscore	5.5	10.8	7.1
Poor rotamers (%)	0.0	0.1	0.0
Ramachandran plot			
Favored (%)	96.2	93.0	92.9
Allowed (%)	3.8	7.0	7.1
Disallowed (%)	0.0	0.0	0.0

	#4 Basal structure – C7 plug (EMDB-51793) (PDB 9H2C)	#5 Apical cap – C2 core (EMDB-51794)	#6 Apical cap - anchor complex (EMDB-51808) (PDB 9H2J)
**Data collection and processing**			
Magnification	64 000	64 000	64 000
Voltage (kV)	300kV	300kV	300kV
Electron exposure (e–/Å^2)	30	30	30
Defocus range (μm)	-1 to -2.2	-1 to -2.2	-1 to -2.2
Pixel size (Å)	1.35	1.35	1.35
Symmetry imposed	C7	C2	C14
Final particle images (no.)	17468	26008	8327
Map resolution (Å)	3.4	6.0	4.7
FSC threshold	0.143	0.143	0.143
**Refinement**			
Model resolution (Å)	3.8		6.2
FSC threshold	0.5		0.5
Model composition			
Non-hydrogen atoms	7191		28102
Protein residues	876		3427
Ligands	0		3
R.m.s. deviations			
Bond lengths (Å)	0.0032		0.0046
Bond angles (°)	0.64		0.90
Validation			
MolProbity score	1.8		2.2
Clashscore	11.2		15.5
Poor rotamers (%)	0.4		0.0
Ramachandran plot			
Favored (%)	96.6		91.3
Allowed (%)	3.4		8.6
Disallowed (%)	0.0		0.2

	#7 Apical cap – C2 plug (EMDB-51803) (PDB 9H2H)	#8 Apical cap – C21 ring (EMDB-51809) (PDB 9H2K)
**Data collection and processing**		
Magnification	64 000	64 000
Voltage (kV)	300kV	300kV
Electron exposure (e–/Å^2)	30	30
Defocus range (μm)	-1 to -2.2	-1 to -2.2
Pixel size (Å)	1.35	1.35
Symmetry imposed	C2	C21
Final particle images (no.)	12477	6390
Map resolution (Å)	6.1	3.5
FSC threshold	0.143	0.143
**Refinement**		
Model resolution (Å)	7.6	3.8
FSC threshold	0.5	0.5
Model composition		
Non-hydrogen atoms	40597	3455
Protein residues	4664	440
Nucleotides	116	0
Ligands	0	0
R.m.s. deviations		
Bond lengths (Å)	0.0044	0.0048
Bond angles (°)	1.00	1.03
Validation		
MolProbity score	2.1	1.5
Clashscore	23.9	7.4
Poor rotamers (%)	0.0	1.3
Ramachandran plot		
Favored (%)	96.1	98.1
Allowed (%)	3.9	1.9
Disallowed (%)	0.0	0.0
